# Emerging Artificial Intelligence Models for Estimating Breslow Thickness from Dermoscopic Images

**DOI:** 10.3390/biomedicines14010097

**Published:** 2026-01-03

**Authors:** Umberto Santaniello, Francois Rosset, Paolo Fava, Francesco Cavallo, Pietro Quaglino, Simone Ribero

**Affiliations:** 1 Section of Dermatology, Department of Medical Sciences, University of Turin, 10126 Turin, Italy; paolo.fava@unito.it (P.F.); francesco.cavallo@unito.it (F.C.); pietro.quaglino@unito.it (P.Q.); simone.ribero@unito.it (S.R.); 2 Department of Dermatology, Beauregard Hospital, Azienda USL della Valle d’Aosta, 11100 Aosta, Italy; francois.rosset@unito.it

**Keywords:** artificial intelligence, deep learning, convolutional neural networks, Breslow thickness, cutaneous melanoma, dermoscopy, machine learning, transfer learning

## Abstract

Breslow thickness (BT) is the most powerful prognostic indicator in cutaneous melanoma, yet histopathological measurement exhibits some limitations such as interobserver variability and diagnostic delays. Preoperative clinical assessment demonstrates 30% misclassification rates. This narrative review synthesizes evidence on deep learning models for non-invasive BT estimation from dermoscopic images. Convolutional neural networks (ResNet, EfficientNet, Vision Transformers) with transfer learning from ImageNet achieve up to 75–79% accuracy and AUC 0.76–0.85 on single-center datasets. Preprocessing techniques (hair removal, color normalization, data augmentation) and interpretability methods (Grad-CAM, LIME) enhance clinical applicability. However, external validation reveals performance degradation. The clinically critical thickness range (0.4–1.0 mm) demonstrates poor discrimination. Significant dataset bias exists: most training data represents lighter skin phototypes, resulting in an underrepresentation of darker skin types. AI models function as complementary decision-support tools rather than replacements for histopathology. Prospective clinical trials validating clinical utility are lacking, and regulatory approval pathways are undefined. Research priorities include diverse public datasets with balanced skin tone representation, the adoption of threshold-weighted loss functions to prioritize accuracy at the 0.8 mm surgical cut-off, multi-institutional external validation, prospective randomized trials, federated learning frameworks, and regulatory engagement. Only rigorous, equitable research can translate AI from proof-of-concept to clinically reliable tools benefiting all melanoma patients.

## 1. Introduction

Cutaneous Melanoma (CM) incidence has escalated globally, with approximately 331700 new cases and 58700 deaths in 2022 [1]. Breslow thickness (BT) remains the most powerful prognostic indicator in CM, impacting on AJCC staging and directly influencing surgical margins, sentinel lymph node biopsy decision, prognosis, and therapeutic implications, according to current guidelines [2,3,4]. Current assessment of BT relies on histopathological examination, precluding preoperative decision-making. In fact, clinical and dermoscopic estimation of tumor depth prior to biopsy is prone to substantial error and interobserver variability, leading to misclassification in up to 30% of cases and potentially to inappropriate management [5]. Preoperative estimation could enable one-step surgery, reducing costs and patient discomfort [6]. In this scenery, deep learning (DL), particularly convolutional neural networks (CNNs), has achieved high accuracy in BT classification using large, annotated datasets [7]. Extending these methods to regression tasks, AI models that predict continuous BT or clinically relevant thresholds (≤1 mm vs. >1 mm) from dermoscopic images were developed [8]. Critical preprocessing steps include hair artifact removal, color normalization, and data augmentation to enhance robustness [9,10]. Transfer learning from ImageNet-pretrained networks accelerates convergence and improves performance on limited medical datasets. Interpretability is addressed through techniques such as Grad-CAM and LIME, which localize salient regions influencing predictions and align AI outputs with histopathological landmarks [11]. Federated learning approaches further bolster generalizability by enabling multi-institutional training without raw data sharing. Despite promising results, challenges remain, including dataset bias, regulatory approval pathways, and the need for prospective clinical validation [12]. This narrative review summarizes current AI models for BT estimation by dermoscopic images, examines their clinical implications, and outlines future research priorities.

## 2. Materials and Methods

A targeted narrative literature review was conducted to identify English-language publications from January 2020 through June 2025 investigating artificial intelligence models for non-invasive BT estimation from dermoscopic images. Multiple databases were searched: PubMed (using MeSH and text terms including “Breslow thickness” AND (“artificial intelligence” OR “deep learning” OR “machine learning” OR “neural network” OR “convolutional neural network”); “melanoma thickness” AND (“CNN” OR “prediction” OR “estimation”); “dermoscopy” AND (“thickness” OR “depth” OR “prognostic”)); arXiv; IEEE Xplore; Scopus; and Google Scholar. Additional studies were identified through manual review of reference lists in key clinical dermatology, medical imaging, and AI journals. Inclusion criteria: research articles or preprints reporting development and/or validation of AI models specifically designed for Breslow thickness estimation or classification from dermoscopic images. Given the historical evolution of AJCC guidelines, studies were included regardless of the specific Breslow threshold used (e.g., 0.75 mm, 0.76 mm, or 0.8 mm), with these differences explicitly noted in the analysis. Data extraction captured study design, dataset characteristics, preprocessing techniques, model architectures, training parameters, validation methodology, performance outcomes, demographic characteristics when reported, and stated limitations. Synthesis was qualitative, identifying trends across architectures, comparing performance, highlighting interpretability advances, and identifying implementation gaps.

## 3. Results and Discussion

### 3.1. Histopathological Breslow Thickness Measurement: The Current Gold Standard and Its Limitations

Histopathological measurement of Breslow thickness, performed on excised melanoma specimens via light microscopy, represents the established gold standard for tumor depth assessment [13]. The technique, first described by Alexander Breslow in 1970, measures the maximum vertical distance from the granular layer of the epidermis (or ulcer base if ulceration is present) to the deepest invasive melanoma cell using a calibrated ocular micrometer at standardized magnification [14]. This measurement directly informs AJCC T-category: pT1a (≤0.8 mm), pT1b (0.8–1.0 mm or ≤0.8 mm with ulceration), stage pT2 (1.0–2.0 mm), pT3 (2.0–4.0 mm), and pT4 (>4.0 mm) [15]. These thresholds directly determine critical management decisions including surgical margin width, sentinel lymph node biopsy (SLNB) indication, and adjuvant therapy consideration [16]. Despite being the reference standard, histopathological BT measurement exhibits some limitations that motivate development of non-invasive alternatives. For example, certain studies have documented substantial interobserver variability in BT measurement among pathologists. A large international population-based study comparing community pathologists with expert dermatopathologists found a kappa statistic of 0.72 for BT categories, indicating “substantial” but imperfect agreement, dropping to moderate (kappa 0.56) for lethal cases [17]. A multicenter reproducibility study evaluating diagnostic concordance in melanocytic proliferations found that diagnoses spanning moderately dysplastic nevi to early-stage invasive melanoma were neither reproducible nor accurate [18]. Pathologists applied an average of 10 different diagnostic terms to identical cases, with 13.2% of cases overinterpreted and 9.2% under interpreted by the initial pathologist relative to expert consensus [18]. These findings suggest that even trained specialists exhibit clinically meaningful disagreement, particularly for lesions near critical thresholds (e.g., 0.8 mm, 1.0 mm) that determine staging and SLNB candidacy. Causes of variability include the following: (1) ambiguity in identifying the granular layer endpoint in cases with regression or ulceration [19,20]; (2) incomplete or inadequate tumor sampling; (3) variation in microscope calibration and measurement technique [21]; (4) subjective interpretation of “deepest invasive cell” in tumors with irregular invasion patterns [22]. Moreover, histopathological BT measurement necessitates surgical biopsy, tissue fixation, embedding, sectioning, staining, and microscopic examination—a process requiring at least 3–10 days depending on institutional workflow. This period, in resource-limited settings, can extend to weeks or months. The direct costs of melanoma biopsy and histopathological assessment vary widely by healthcare system but typically range from USD 150 to 500 per procedure in the United States [23]. Additional burden is associated with indirect costs (patient time, transportation, loss of productivity), which are particularly relevant for underserved populations with limited healthcare access. In sum, the limitations of histopathological BT measurement (interobserver variability, procedural delays, economic costs, patient burden, and sampling error) provide a rationale for developing accurate, non-invasive, objective estimation methods. In this scenery, AI models analyzing dermoscopic images offer potential to address these limitations by providing instantaneous, reproducible, cost-effective depth estimates at the point of clinical evaluation, enabling earlier risk stratification and treatment planning while reducing unnecessary biopsies.

### 3.2. Artificial Intelligence in Medical Imaging: A Paradigm Shift

Artificial intelligence (AI) encompasses computational systems designed to perform tasks traditionally requiring human cognition, including pattern recognition, decision-making, and predictive modeling [24]. Within AI, machine learning (ML) represents a fundamental model wherein algorithms learn to make predictions from data without explicit programming of decision rules. Rather than manually coding diagnostic criteria, ML algorithms automatically identify statistical patterns correlating input features (dermoscopic image characteristics) with target outputs (BT measurements) [24]. Traditional ML approaches required extensive domain expertise to manually engineer features from raw data. In contrast, modern DL represents a transformative subset of ML that eliminates manual feature engineering by automatically discovering hierarchical representations directly from raw data [24]. The convergence of DL algorithms, large-scale datasets, and computational advances has catalyzed a revolution in medical image analysis [25]. The landmark 2017 publication by Esteva and colleagues demonstrated that a CNN trained on 129,450 clinical images achieved dermatologist-level performance in classifying skin cancer, with sensitivity–specificity trade-offs comparable to 21 board-certified dermatologists [26]. This seminal work utilized an Inception v3 architecture pretrained on ImageNet, subsequently fine-tuned for skin lesion classification, establishing a foundational approach for subsequent investigations [27]. CNNs excel at automatically learning hierarchical feature representations from raw image data, avoiding the need for hand-crafted features [28]. Multiple studies have subsequently confirmed that deep learning models can match or exceed dermatologist performance in melanoma detection tasks [29,30].

### 3.3. CNN Architectures for Melanoma Analysis

**Figure 1:**
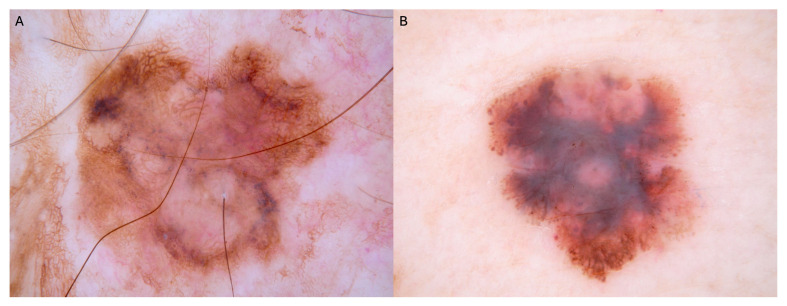
Representative dermoscopic images from the ISIC Archive showing melanomas with different Breslow thicknesses. (**A**) Melanoma in situ showing asymmetry, peppering, structureless areas, irregular borders, and atypical pigment network. Melanomas in situ typically present with fewer colors (1–2 colors), absence of shiny white structures, absence of blue-white veil, and lack of milky-red areas, reflecting their confinement to the epidermis without dermal invasion. (**B**) Invasive melanoma (Breslow thickness 1.03 mm), exhibiting multicomponent pattern, blue-gray veil, and milky-red areas, indicating dermal invasion. Both images were obtained from the International Skin Imaging Collaboration (ISIC) Archive (Creative Commons CC0 license), the largest publicly available collection of quality-controlled dermoscopic images used for developing and training AI algorithms.

CNNs revolutionized medical image analysis by automatically learning hierarchical image features without manual engineering [31]. Unlike traditional machine learning approaches requiring hand-crafted features, CNNs comprise multiple layers that progressively extract increasingly abstract features: early convolutional layers capture low-level features (edges, textures), intermediate layers combine these into mid-level structures (characteristic patterns), and deeper layers synthesize high-level semantic features (diagnostic hallmarks). This hierarchical learning enables CNNs to achieve superior performance on complex visual tasks, including medical image classification [32]. Several proven architectures have been adapted for BT estimation in CM; the following architectures are highlighted based on their recurrent application and superior performance in the reviewed literature:

- ResNets (Residual Networks): introduced by He et al., they employ skip connections that facilitate training of exceptionally deep architectures (50, 101, 152 layers) by mitigating vanishing gradient problems [33]. ResNet variants (ResNetV2, ResNet50, ResNet152) have demonstrated strong performance in melanoma classification tasks [34,35]. For Breslow thickness prediction, ResNetV2 achieved AUC-ROC of 0.76 for thickness comparisons [8].
- EfficientNet: achieves state-of-the-art accuracy with improved computational efficiency by optimizing the balance between network depth, width, and resolution [36]. EfficientNetB6 recorded the highest diagnostic accuracy (75%) for BT classification (<0.8 mm vs. ≥0.8 mm), outperforming dermatologist groups [8]. Himel et al. report EfficientNetB4 achieving 87.9% accuracy on the HAM10000 dataset (a publicly available collection of 10015 dermoscopic images) for multi-class skin lesion classification [37].
- InceptionV3: this architecture employs multiple parallel convolutional operations at different scales, enabling efficient extraction of diagnostic morphologic features across different magnification levels—a characteristic particularly relevant to dermoscopic image interpretation. For BT assessment, InceptionV3 achieved AUC-ROC of 0.75, demonstrating competitive performance [8]. The Esteva landmark study utilized InceptionV3 as a base architecture [26].
- DenseNet: Dense Convolutional Networks connect each layer to every subsequent layer, promoting feature reuse and gradient flow [38]. DenseNet121 and DenseNet201 have achieved over 95% accuracy on melanoma detection tasks using HAM10000 and ISIC datasets (Figure 1) [11,39]. DenseNet-201 achieved 82.9% accuracy on seven-class HAM10000 classification [37].
- Vision Transformers (ViTs): transformer architectures originally developed for natural language processing that have been adapted for image analysis [40]. Vision transformers divide images into patches and process them using self-attention mechanisms, potentially capturing long-range morphologic dependencies better than CNNs [41]. Early applications to skin lesion classification show promise, with modified ViT models achieving competitive performance with CNN architectures [42].
- ConvNeXt: introduced by Liu et al., it modernizes the ResNet design with architectural innovations inspired by ViTs, within a purely convolutional framework [43]. Early dermatology applications show its promise for melanoma diagnosis and continuous BT regression [44,45].
- RegNet: proposed by Radosavovic et al., it introduces a design space that systematizes scaling of depth, width, and group convolution parameters to improve both accuracy and efficiency [46]. RegNet models exhibit competitive performance in medical imaging and outperform ResNet families. In dermatology, RegNet-based (Regdisnet) approaches have demonstrated robust accuracy (93.21%) for melanoma detection [47].

### 3.4. Image Preprocessing Techniques and Transfer Learning

Effective preprocessing of images is critical for robust model performance. For example, hair overlaying lesions can obscure diagnostic features and confound automated analysis. Morphological operations, digital hair removal filters, and DL-based techniques address this challenge [9,48]. Recent advances include synthetic hair benchmark datasets for evaluating removal algorithms [10]. Moreover, variability in imaging equipment, lighting conditions, and camera settings introduces color inconsistencies. Histogram equalization, color constancy algorithms, and color normalization standardize images across different acquisition systems [49,50]. To artificially expand limited training datasets and improve model robustness, data augmentation techniques are essential. Data augmentation techniques include geometric transformations (rotation, translation, scaling, flipping); color space perturbations (brightness, contrast, saturation adjustments); elastic deformations; and random cropping [51,52]. These techniques yield substantial performance gains, with reported accuracy increases exceeding 12% for CM classification [53]. Finally, image standardization entails resizing images to consistent dimensions (typically 224 × 224 or 299 × 299 pixels, aligning with ImageNet preprocessing), normalizing pixel values to the range [0, 1], or standardizing them to have a mean of zero and a variance of one [54]. Given the relatively limited size of medical imaging datasets compared to natural image datasets, transfer learning has become a cornerstone strategy [55]. This approach involves pretraining on ImageNet, a dataset comprising 1.2 million images, which enables the learning of generalizable low-level features such as edges, textures, and shapes. Subsequently, the model undergoes fine-tuning on dermoscopic images, allowing it to adapt its high-level features to the specific patterns associated with skin lesions. This method offers several benefits, including accelerated convergence, improved performance on small datasets, and reduced risk of overfitting [56]. Transfer learning from ImageNet-pretrained networks significantly outperforms models trained exclusively on dermatologic image datasets [57]. The limited availability of large-scale dermoscopic datasets with histopathologically confirmed BT measurements makes transfer learning particularly critical for thickness estimation tasks [58].

### 3.5. Current AI Model for Breslow Thickness Estimation

As one of the first studies to apply ML to melanoma thickness classification, Sáez et al. (2015) [59] proposed a computerized system using 250 dermoscopic images (167 thin <0.76 mm, 54 intermediate 0.76–1.5 mm, 29 thick >1.5 mm). Feature extraction included 81 descriptors: shape, color, pigment network features, and texture analysis. For binary classification (thin vs. thick), the model achieved 77.6% accuracy. For three-class classification, the model obtained 68.4% overall accuracy but was outperformed by ordinal classification methods (55.2% accuracy on the worst-classified thick category with mean absolute error <0.5). Ordinal classification achieved better performance balance across all classes compared to nominal classifiers. The study was limited by its small, single-institution dataset and non-standardized image acquisition [59]. A pioneering study by Jaworek-Korjakowska et al. (2019) [60] introduced a DL approach for preoperative BT estimation using Transfer Learning with a VGG-19 architecture. The model was trained on 244 dermoscopic images from the Interactive Atlas of Dermoscopy, classifying lesions into three thickness categories: thin (<0.75 mm), intermediate (0.76–1.5 mm), and thick (>1.5 mm). To address data scarcity and class imbalance, the authors applied SMOTE (Synthetic Minority Over-sampling Technique) for data augmentation. This methodology achieved an overall accuracy of 87.2% [60]. Hernández-Rodríguez JC et al. (2023) developed and validated three deep transfer learning algorithms to predict melanoma in situ versus invasive melanoma and classify Breslow thickness (<0.8 mm vs. ≥0.8 mm) [8]. The study utilized a dataset of 1315 dermoscopic images of histopathologically confirmed melanomas from the Virgen del Rocío University Hospital and open repositories (ISIC archive, Polesie et al. dataset). EfficientNetB6 achieved the highest diagnostic accuracy for in situ versus invasive melanoma (61%, AUC 0.54) but was outperformed by 10 dermatologists (mean accuracy 64%, AUC 0.64, κ = 0.46). For BT comparison, ResNetV2 (AUC 0.76) and InceptionV3 (AUC 0.75) significantly outperformed the dermatologist group (mean AUC 0.70, accuracy 69%, κ = 0.35), while EfficientNetB6 achieved the highest accuracy (75%, AUC 0.69). Gradient-CAM visualization highlighted regions influencing model predictions. The study concluded that deep transfer learning algorithms represent useful ancillary aids for preoperative thickness stratification, particularly for the 0.8 mm threshold guiding SLNB decisions, though standardization of dermoscopic imaging acquisition is necessary for optimal CNN performance [8]. Szijártó et al. (2023) [61] proposed a non-invasive system for categorizing BT into three classes (<0.76 mm, 0.76–1.5 mm, >1.5 mm) using an EfficientNet-B4 CNN trained on 247 dermoscopic images from the Derm7pt dataset. The methodology incorporated transfer learning, aggressive data augmentation, and weighted loss functions, validating performance via five-fold cross-validation. For binary classification (thin vs. thick), the model achieved an accuracy of 77.6% and an AUC of 0.83. For the three-class task, it attained an overall accuracy of 71.6% and a multiclass AUC of 0.834, resulting in a final balanced accuracy across folds of 70.8%, outperforming prior logistic regression and ordinal methods (LIPU: 44.9%, KDLOR: 59.9%). The authors also re-evaluated Jaworek-Korjakowska et al. [60], showing their 87.2% accuracy resulted from data leakage due to improper SMOTE application. The corrected model’s accuracy dropped to 52.5%, underscoring the necessity of rigorous validation protocols to avoid biases. Performance projections suggest that expanding the training dataset could significantly further improve diagnostic precision, validating DL potential for preoperative tumor staging [61]. Polesie et al. (2022) [5] compared human evaluators (n = 438) against two CNNs for estimating BT in 1456 dermoscopic images. Collective human accuracy for three-category classification was 56.4% (rising to 66.9% with majority voting). In binary classification (≤1 mm vs. >1 mm), humans achieved 85.9% accuracy for thin lesions but only 70.8% for thick ones. A pretrained ResNet-50 model (AUC = 0.83) performed comparably to collective human judgment (AUC = 0.85), while a de novo CNN (AUC = 0.80) proved significantly inferior. Notably, accuracy was poorest in the intermediate 1–2 mm range (39% correct classification), indicating an inherent “gray zone” in dermoscopy. Reader experience did not significantly impact results, suggesting objective imaging limitations rather than expertise gaps. The authors concluded that current CNNs are insufficient for independent clinical use without prospective validation [5]. Gillstedt et al. (2022) [62] evaluated a multimodal CNN (clinical plus dermoscopic images) against six dermatologists for distinguishing invasive from in situ melanomas (n = 1578). The CNN achieved an AUC of 0.73, significantly underperforming compared to collective human assessment (AUC = 0.80). While the model excelled at identifying thick melanomas >1.0 mm (AUC = 0.93)—though it was still slightly outperformed by dermatologists in this category (AUC = 0.97)—its accuracy dropped sharply for thin invasive lesions ≤1.0 mm (AUC = 0.64), a domain where dermatologists remained superior (AUC = 0.74). Interestingly, integrating clinical images did not improve performance over dermoscopy alone (AUC = 0.72), highlighting dermoscopy’s dominant discriminatory value. Specific features like shiny white lines and atypical blue-white structures showed high specificity (>90%) for invasion. The study concludes that despite AI advancements, expert human judgment remains superior, particularly for early-stage invasive lesions, necessitating further validation before clinical adoption [62]. Dominguez-Morales et al. (2024) [7] introduced an ensemble-based multi-teacher knowledge distillation approach combined with semi-supervised CNNs to classify melanoma Breslow thickness (BT) and distinguish in situ from invasive lesions. Utilizing heterogeneous datasets (n = 1449 labeled images) and an external test set (n = 153), the study compared supervised learning against a semi-supervised framework where teacher models pseudo-annotated unlabeled data. Results consistently favored the semi-supervised approach. For binary BT classification (<0.8 mm vs. ≥0.8 mm), the semi-supervised model achieved superior performance (AUC 0.82 vs. 0.77 for supervised), representing a 5% improvement. Similarly, discrimination between in situ and invasive melanoma improved significantly (AUC 0.81 vs. 0.75), as did multiclass analysis (AUC 0.76 vs. 0.72). The authors concluded that leveraging unlabeled data through ensemble knowledge distillation enhances diagnostic accuracy, outperforming traditional supervised methods and positioning this semi-supervised architecture as a viable clinical decision-support tool [7]. A cross-study synthesis reveals a nuanced dichotomy between algorithmic metrics and clinical utility. While some studies, such as Hernández-Rodríguez et al., report AI architectures (e.g., ResNetV2) outperforming dermatologists in binary classification (AUC 0.76 vs. 0.70) [8], larger validation cohorts paint a different picture. Polesie et al. and Gillstedt et al. demonstrated that collective human intelligence retains superior specificity, particularly for thin invasive melanomas (≤1.0 mm), where CNN performance drops significantly (AUC 0.64) compared to experts (AUC 0.74) [5,62]. Crucially, this comparison highlights a structural flaw in current AI training strategies: high global performance metrics (e.g., AUC > 0.80) are often driven by the model’s ability to easily classify obvious extremes (very thin or very thick tumors), masking substantial failures in the clinically decisive ‘gray zone’ (0.75–1.0 mm). Consequently, the ‘smart’ evolution of these tools should not merely pursue higher overall accuracy, but rather adopt threshold-weighted loss functions that specifically penalize prediction errors around the 0.8 mm surgical cut-off, aligning algorithmic penalties with the clinical cost of mis-staging. Table 1 provides a comparative overview of the main deep learning studies focused on melanoma BT classification.

### 3.6. Explainability and Interpretability

DL models have demonstrated high accuracy in dermatologic image analysis, yet their internal mechanisms remain largely cloudy to clinicians. This “black box” nature represents a significant barrier to clinical implementation of medical AI systems [63]. The lack of interpretability directly affects trust and adoption: clinicians are less inclined to rely on systems whose diagnostic reasoning they cannot understand. Moreover, without insight into how a model arrives at its predictions, error analysis becomes difficult. From a regulatory perspective, transparency is increasingly required by medical authorities, and medico-legal accountability requires that decision pathways be interpretable and traceable [64,65]. To address these limitations, several interpretability techniques have been developed. Gradient-weighted Class Activation Mapping (Grad-CAM) provides visual explanations by highlighting the regions of an image that most strongly influence a model’s decision [66]. In CM diagnosis, Grad-CAM has been applied to visualize the “attention” of neural networks, verifying whether the algorithm focuses on clinically meaningful dermoscopic features and identifying potential artifacts that might bias predictions [67]. Advanced variants like Grad-CAM++, Score-CAM, and Eigen-CAM further refine these maps [68]. Another major interpretability framework is Local Interpretable Model-agnostic Explanations (LIME), which seeks to understand model behavior by approximating the decision of complex networks with simpler, more interpretable models. LIME works to identify which regions are most critical for classification [69,70]. Giavina Bianchi M et al. (2023) evaluated multiple visualization methods, including Grad-CAM, Grad-CAM++, Score-CAM, Eigen-CAM, and LIME, assessing their alignment with established clinical diagnostic criteria such as asymmetry, border irregularity, and color heterogeneity [68]. Their results revealed significant variability among methods, underscoring the necessity of rigorous validation of explainability outputs against clinical ground truth [68]. Similarly, Naseri et al. (2025) conducted a systematic review on diagnosis and prognosis of melanoma using ML and DL, emphasizing the importance of integrating interpretable AI techniques into clinical workflows to provide visual feedback, thereby enhancing both transparency and clinician confidence in ML models [11]. Attention mechanisms offer intrinsic transparency by designating which regions or attributes most influence predictions [71]. These interpretability strategies represent a crucial bridge between algorithmic precision and clinical trust. Their validation will be essential for transforming AI from a diagnostic assistant into a fully integrated component of dermatologic decision-making.

### 3.7. Clinical Application and Workflow Integration

AI models capable of estimating BT from dermoscopic images are emerging as promising tools for dermatologic oncology. Their utility extends across the continuum of melanoma care, from preoperative decision-making to triage, workflow optimization, and post-histopathological validation. An important role for these systems is triage and prioritization. Automated thickness prediction could help identify high-risk lesions that warrant urgent biopsy, particularly in settings where access to dermatologic expertise or pathology services is limited. In teledermatology frameworks, AI triage algorithms could flag concerning cases for expedited evaluation, streamlining referral pathways and optimizing the allocation of clinical resources [72]. One of the most interesting applications lies in preoperative planning, where AI-assisted BT estimations can inform initial surgical management decisions. Predicting whether a lesion is likely to exceed clinically relevant BT thresholds (e.g., 0.8 mm) allows surgeons to select appropriate excision margins and assess the need for SLNB before histopathological confirmation. Furthermore, early AI-derived information on likely prognosis can support patient counseling at the time of diagnosis [73]. In addition, AI tools may serve as decision-support systems that complement, rather than replace, clinician judgment. By providing quantitative, reproducible assessments of BT, these models can act as a “second opinion,” helping reduce inter-observer variability and enhancing diagnostic confidence, especially among less experienced practitioners [74]. Implementation in clinical practice requires standardized image acquisition protocols, followed by automated preprocessing steps to prepare dermoscopic images for analysis. Importantly, interpretability features should highlight the image regions that most influenced the prediction, facilitating clinician trust and error analysis. Integration with electronic health records would allow automatic storage and reporting of results, ensuring traceability. Cloud-based infrastructure further expands accessibility: clinicians could access AI predictions through secure web platforms or mobile applications, making deployment feasible in diverse healthcare environments [75]. Basically, AI-derived BT estimates are not intended to replace histopathology, which remains the diagnostic gold standard. Instead, these predictions should be viewed as complementary, providing preoperative guidance and enabling postoperative comparison with definitive histopathologic results. Such comparisons can serve as ongoing calibration for AI systems, identifying systematic biases and guiding continuous model improvement. Despite these advances, real-world implementation presents several challenges. Future validation studies must explicitly measure these workflow impacts, specifically quantifying the additional time required for image analysis and assessing the risk of automation bias in borderline cases, to confirm practical utility.

### 3.8. Challenges and Limitations

AI in melanoma diagnosis and BT estimation faces significant technical, clinical, and ethical challenges. A major concern is dataset bias and representation. The vast majority of large-scale dermatologic image datasets are composed of individuals with Fitzpatrick skin types I to III. As a result, AI systems trained on such datasets tend to perform poorly when applied to patients with darker skin (Fitzpatrick IV–VI) [76,77]. Beyond skin tone, datasets are also shaped by geographic and institutional biases. Data collected in a single region or medical center may reflect specific epidemiologic patterns, dermoscopic practices, or device characteristics that limit generalizability [78]. Additionally, public datasets often suffer from lesion-type imbalance, with overrepresentation of common subtypes such as superficial spreading melanoma and underrepresentation of rarer but clinically significant forms [79]. Another important limitation is the scarcity of datasets with reliable BT annotations. Consequently, model complexity and generalization are limited, and the field lacks the kind of large, annotated benchmarks that boosted progress in other domains of medical imaging. Issues in validation methodology further complicate interpretation of AI performance. Many published studies depend solely on internal validation. This approach risks data leakage and tends to overestimate model performance by failing to capture real-world variability. True robustness requires external validation on independent datasets collected from different institutions, devices, and patient populations [80]. Moreover, the field remains dominated by retrospective analyses. Prospective clinical trials, which are necessary to evaluate how AI impacts diagnostic decisions and patient outcomes in practice, are still lacking. A further barrier lies in interpretability and clinical trust. Even with tools like Grad-CAM and LIME offering visual explanations, clinicians remain cautious of opaque “black box” predictions [81]. Surveys indicate that clinicians place high value on understanding the rationale behind AI-generated diagnoses and may disbelieve systems that cannot provide transparent reasoning [82,83]. Building trust requires demonstrating that explainability maps correspond to recognized dermoscopic criteria, that model failures are predictable and interpretable, and that AI augmentation genuinely improves clinical performance rather than adding confusion. Reimbursement pathways present additional challenges for AI-assisted diagnostics; healthcare systems require solid evidence of cost-effectiveness and demonstrable clinical utility before establishing a refund [84]. Regulatory approval represents another significant hurdle. As ‘Software as a Medical Device’ (SaMD), these algorithms must undergo rigorous clearance (e.g., FDA, EU MDR), proving not just technical accuracy but clinical safety and effectiveness. To date, cost-effectiveness analyses quantifying the economic impact of AI-guided staging, balancing the savings of reduced biopsies against the liability of missed thick melanomas, are notably absent from the literature. Finally, ethical considerations pervade every stage of AI deployment. Questions of accountability remain unresolved: if an AI system contributes to a diagnostic error, determining whether the clinician, developer, or institution bears legal responsibility is far from straightforward [85]. Informed consent also takes on new dimensions, as patients have the right to know when and how AI tools influence their diagnosis and management. Above all, ensuring health equity is paramount. Without deliberate effort to design, validate, and deploy models inclusively, AI risks amplifying existing disparities rather than reducing them [86]. The translation of AI-based melanoma thickness estimation from research to real-world use depends not only on technical accuracy but also on equitable data representation, robust validation, interpretability, and clear regulatory and ethical frameworks. Addressing these multidimensional challenges will determine whether AI in dermatology evolves into a reliable clinical ally or remains confined to proof-of-concept studies.

### 3.9. Future Directions

**Figure 2:**
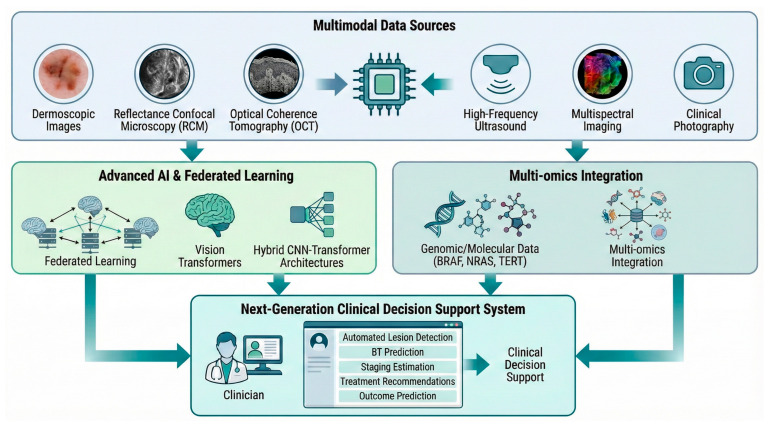
Conceptual framework for a future integrated AI ecosystem for melanoma clinical decision support. Multimodal Data Sources (**Top Panel**): Acquisition of diverse prospective imaging and clinical data inputs, comprising dermoscopy, reflectance confocal microscopy (RCM), optical coherence tomography (OCT), high-frequency ultrasound, multispectral imaging, and clinical photography. Advanced Processing and Multi-omics Integration (**Middle Panels**): Parallel processing streams utilizing cutting-edge AI architectures, including Vision Transformers and Hybrid CNN-Transformer networks, trained via decentralized Federated Learning paradigms (**left**). Concurrently, genomic and molecular biomarkers (e.g., BRAF, NRAS, TERT mutations) are incorporated through Multi-omics Integration strategies (**right**). Next-Generation CDSS (**Bottom Panel**): Convergence of these processed data streams into a Clinical Decision Support System interface. The clinician is provided with integrated, automated outputs for lesion detection, Breslow thickness (BT) prediction, staging estimation, guideline-aligned treatment recommendations, and outcome prediction.

The future of artificial intelligence in BT estimation is heading toward integration, collaboration, and clinical reliability (Figure 2). One of the most promising directions lies in multimodal approaches, where dermoscopic data are combined with other modalities to enhance diagnostic accuracy. The integration of dermoscopy with clinical photography and advanced imaging techniques such as reflectance confocal microscopy (RCM), optical coherence tomography (OCT), High-Frequency Ultrasound, or multispectral imaging may provide complementary depth-resolved and structural information, supporting more accurate BT estimation [73,87,88]. Equally important is the emergence of federated learning, a decentralized training paradigm that allows multiple institutions to collaboratively develop AI models without sharing raw patient data [89]. Each site trains a local model, and model parameters are shared and aggregated. This approach enhances generalizability by leveraging diverse datasets across geographic and demographic boundaries. Federated learning has already demonstrated feasibility in melanoma classification, achieving accuracies of 90.02% on HAM10000 and 86.03% on ISIC 2019 [90]. Extending such frameworks to BT prediction could dramatically increase dataset diversity and scalability while addressing current limitations in data heterogeneity and bias. From a computational standpoint, Vision Transformers and hybrid CNN-transformer architectures represent a major paradigm shift in image understanding. These models, adapted from natural language processing, excel at capturing long-range spatial dependencies and contextual information within dermoscopic images. Early studies applying transformer-based models to melanoma classification have shown promising results, indicating strong potential for future applications in continuous variable prediction tasks such as BT estimation [91]. The long-term evolution of AI in melanoma care will also depend on integration with genomic and molecular data. Combining dermoscopic AI-based thickness predictions with genetic (e.g., BRAF, NRAS, TERT mutations) and other prognostic biomarkers may enable comprehensive and individualized risk stratification. Such multi-omics integration could ultimately refine prognostication beyond the histological measure of thickness alone [92]. The convergence of these advances is expected to yield a next generation of “clinical decision support systems”. These systems may integrate automated lesion detection, BT prediction, staging estimation, treatment recommendations aligned with international clinical guidelines, and outcome prediction [93]. The trajectory of AI in dermatology is rapidly evolving from image-centric experimentation to multimodal, adaptive, and clinically integrated systems. Through federated collaboration, self-improving architectures, and biologically integrated models, AI is set to support, and not replace, expert clinical decisions in melanoma diagnosis and management.

## 4. Conclusions

AI, particularly DL using CNNs, has shown strong potential for non-invasive estimation of BT from dermoscopic images, with models like ResNet and EfficientNet achieving 75–79% accuracy and AUCs of 0.76–0.85 in controlled settings. Critical preprocessing strategies enhance model reliability, while explainability tools like Grad-CAM and LIME help mitigate the “black box” concern, promoting transparency and clinician confidence. Nevertheless, the path to clinical translation remains challenging. The scarcity of large datasets, the underrepresentation of darker skin phototypes and rare melanoma subtypes, and lack of external validation and prospective clinical trials continue to delay implementation in real-life settings. Current MAE values of ~1 mm remain significant, especially given current AJCC thresholds. Future progress requires not only large, diverse, and balanced datasets via multi-institutional collaboration and federated learning, but also the implementation of threshold-weighted loss functions to explicitly minimize prediction errors around the critical 0.8 mm surgical cut-off. Advances in multimodal integration—combining dermoscopic imaging with clinical photographs, patient metadata, molecular profiles, and advanced imaging techniques such as RCM and OCT—are likely to enhance predictive accuracy and clinical applicability. In conclusion, AI-based BT prediction should be viewed as a complementary preoperative tool for surgical planning and triage rather than a substitute for histopathology. Achieving safe and equitable implementation will demand close collaboration among dermatologists, dermatopathologists, AI scientists, regulators, payers, and patients. As datasets expand and algorithms mature, non-invasive AI-driven BT estimation may become a standard in melanoma management, enhancing diagnostic precision, optimizing surgical planning, and improving patient outcomes.

## Figures and Tables

**Table 1:** Comparative summary of key DL studies for BT classification.

Study	Image Modality	Classification Task	Best Model/Method	Primary Performance Metric	Best Result
Nogales et al. [44]	Dermoscopy only	Binary: thin (<0.76 mm) vs. thick (≥0.76 mm)	ConvNext transfer learning with Focal Loss	Accuracy 79%, Recall 0.79, F1 0.67	Accuracy 79%, Recall 0.79, F1-score 0.67; R^2^ = 0.25 overall; zone 0.4–1.0 mm R^2^ = 0.08
Szijártó et al. [61]	Dermoscopy only	Binary: thin (<0.76 mm) vs. thick (≥0.76 mm); Multiclass: <0.76 mm vs. 0.76–1.5 mm vs. >1.5 mm	EfficientNet-B4 with transfer learning, weighted class training, data augmentation, 5-fold cross-validation	Accuracy, Balanced Accuracy, ROC-AUC	Binary: Accuracy 77.6%, Balanced Accuracy 70.8%, AUC 0.83; Multiclass (3-class): Accuracy 71.6%, Balanced Accuracy 70.8%, AUC 0.834
Polesie et al. [5]	Dermoscopy only	3-class (MIS, ≤1.0 mm, >1.0 mm) and binary (thin vs. thick)	Collective reader assessment (majority voting)	3-class AUC 0.85; Binary: thin 85.9%, thick 70.8%	Readers AUC 0.85 >> de novo CNN AUC 0.80; no experience effect (*p* = 0.35)
Hernández-Rodríguez et al. [8]	Dermoscopy only	In situ vs. invasive and Breslow <0.8 mm vs. ≥0.8 mm	ResNetV2 and InceptionV3 for thickness; EfficientNetB6 for invasiveness	ResNetV2 AUC 0.76, InceptionV3 0.75; EfficientNetB6 61% accuracy	For thickness: ResNetV2 AUC 0.76 >> dermatologists 0.70; for invasiveness: EfficientNetB6 61% << dermatologists 64%
Gillstedt et al. [62]	Clinical close-up + dermoscopy	Binary: in situ vs. invasive	De novo CNN (6 + 7 conv layers)	CNN AUC 0.73 vs. dermatologists AUC 0.80	Thick (>1.0 mm): CNN AUC 0.93 vs. 0.97; Thin (≤1.0 mm): CNN AUC 0.64 vs. dermatologists 0.74

## Data Availability

No new data were created or analyzed in this study. Data sharing is not applicable to this article.

## Abbreviations

The following abbreviations are used in this manuscript:

CM	Cutaneous Melanoma
AI	Artificial Intelligence
ML	Machine Learning
DL	Deep Learning
BT	Breslow thickness
RCM	Reflectance Confocal Microscopy
OCT	Optical coherence tomography
MAE	Mean absolute error
AUC	Area under the curve
AJCC	American Joint Committee on Cancer
TNM	Tumor Node Metastasis
ISIC	International Skin Imaging Collaboration
Grad-CAM	Gradient-weighted Class Activation Mapping
CDSS	Clinical Decision Support System
